# Impact of COVID-19 Infections among Unvaccinated Patients with Congenital Heart Disease: Results of a Nationwide Analysis in the First Phase of the Pandemic

**DOI:** 10.3390/jcm13051282

**Published:** 2024-02-24

**Authors:** Alicia Jeanette Fischer, Alina Ruth Hellmann, Gerhard-Paul Diller, Maarja Maser, Carsten Szardenings, Ursula Marschall, Ulrike Bauer, Helmut Baumgartner, Astrid Elisabeth Lammers

**Affiliations:** 1 Department of Cardiology III—Adult Congenital and Valvular Heart Disease, University Hospital Muenster, 48149 Muenster, Germany; alicia.fischer@ukmuenster.de (A.J.F.); helmut.baumgartner@ukmuenster.de (H.B.);; 2 Department of Cardiac Surgery, Tartu University Hospital, 50406 Tartu, Estonia; maarja.maser@kliinikum.ee; 3 Institute of Biostatistics and Clinical Research, University Hospital Muenster, 48149 Muenster, Germany; 4 Department of Medicine and Health Services Research, BARMER Health Insurance, Lichtscheider Strasse 89, 42285 Wuppertal, Germany; ursula.marschall@barmer.de; 5 National Register for Congenital Heart Defects, Competence Network for Congenital Heart Defects, 13353 Berlin, Germany; ubauer@kompetenznetz-ahf.de; 6 Department of Pediatric Cardiology, University Hospital Muenster, 48149 Muenster, Germany

**Keywords:** congenital heart disease, pneumonia, COVID-19, virus diseases, respiratory tract infection

## Abstract

**Background**: The outcome data and predictors for mortality among patients with congenital heart disease (CHD) affected by COVID-19 are limited. A more detailed understanding may aid in implementing targeted prevention measures in potential future pandemic events. **Methods**: Based on nationwide administrative health insurance data, all the recorded in-hospital cases of patients with CHD with COVID-19 in 2020 were analyzed. The demographics, treatment details, as well as 30-day mortality rate were assessed. The associations of the patients’ characteristics with death were assessed using multivariable logistic regression analysis. **Results**: Overall, 403 patients with CHD were treated in- hospital for COVID-19 in 2020. Of these, 338 patients presented with virus detection but no pneumonia whilst, 65 patients suffered from associated pneumonia. The cohort of patients with pneumonia was older (*p* = 0.04) and presented with more cardiovascular comorbidities such as diabetes mellitus (*p* = 0.08), although this parameter did not reach a statistically significant difference. The 30-day mortality rate was associated with highly complex CHD (odds ratio (OR) 7.81, *p* = 0.04) and advanced age (OR 2.99 per 10 years, *p* = 0.03). No child died of COVID-related pneumonia in our dataset. **Conclusions**: COVID-19 infection with associated pneumonia chiefly affected the older patients with CHD. Age and the complexity of CHD were identified as additional predictors of mortality. These aspects might be helpful to retrospectively audit the recommendations and guide health politics during future pandemic events.

## 1. Introduction

When severe acute respiratory syndrome coronavirus 2 (SARS-CoV-2) spread globally in 2019, it led to millions of infections and deaths [1,2]. Clinically, infection was, however, not necessarily associated with a severe disease course. Its presentation varied widely, ranging from entirely asymptomatic individuals to those with respiratory or multi-organ failure, or in the patient’s death in many cases [3]. When affected by an infection, the patients were at risk of cardiac events, most likely due to endothelial dysfunction caused by the interplay between inflammation and thrombosis [4]. In non-selective analyses, up to 80% of predominantly younger patients with coronavirus disease 2019 (COVID-19) presented with mild symptoms only. By contrast, only 5% of the patients in these series were reported to have experienced a severe disease course [5]. For the patients with congenital heart disease (CHD), infectious diseases such as viral pneumonia are generally associated with an increased morbidity and mortality [6]. The potential predictors for a poor outcome in CHD after COVID-19 infection were obviously unknown at the beginning of the pandemic. Consequently, the management strategies of COVID-19 infection among patients with CHD were predominantly extrapolated from the data on patients with cardiovascular diseases who have been found to be at a higher risk of a severe course and increased mortality [7]. The rationale behind using non-congenital patients’ data was that the patients with CHD were considered patients with underlying cardiovascular disease, and thus, were expected to be specifically at risk from a cardiovascular standpoint, despite their young age [8]. Limited knowledge about the virus rendered the targeted identification of patients at risk impossible and led to (in hindsight) potentially extensive primary preventive measures [9] with associated psychological and socioeconomic consequences [10]. Specifically, children’s and adolescents’ mental health was impacted by social distancing and schools closing [11]. The optimal individualized risk stratification approach to treating unvaccinated and unexposed patients with CHD with COVID-19 by implementing targeted primary prevention measures remains unclear until today. This is due to the obvious fact that no randomized controlled data on the topic can be derived for ethical and logistic reasons. Nevertheless, observational data acquired from experiences during the COVID-19 pandemic, however, may aid in preparation for fatalities in potential future pandemic events. Our study aims to give an overview of the patients with CHD that suffered with COVID-19 infection based on unselected data. The patients were divided in patients with COVID-19 infection only and those with associated pneumonia. A specific focus was placed on the patients’ age.

## 2. Materials and Methods

### 2.1. Study Design

Data were acquired retrospectively from the administrative reimbursement dataset of the BARMER health insurance company (≈9.2 million insured individuals of all ages). In the health insurance dataset, all diagnoses were documented using a standardized nationwide coding system, the German Modification of the International Statistical Classification of Diseases and Related Health Problems, 10th Revision (ICD-10-GM). Medical procedures were documented using the German Procedure Classification (OPS) codes. Patients’ characteristics and comorbidities were assessed based on in-hospital diagnostic and procedural codes (for a full list of ICD-10-GM, Anatomical Therapeutic Chemical Classification System (ATC) and OPS procedure codes used in this study, see Table S1, online supplement). All the patients registered in the dataset in 2020 with a diagnosis of CHD were identified. The severity of CHD was graded as mild, moderate, or severe according to the current guidelines [12] (see Table S2, online supplement). COVID-related pneumonia was defined based on the ICD-10 codes J09, J 10.0, J11.0, and J12.0 combined with the following COVID-19-related ICD-10 codes: U07.1! or U07.2!. The diagnosis of COVID-19 was defined as the index event. For inclusion, the patients were either required to present with typical symptoms of COVID-19 or with a positive test result. The patients were allocated to one of two cohorts: either (1) isolated COVID-19 virus, or (2) disease with associated pneumonia. Descriptive data are provided for both cohorts. Furthermore, the cohorts were subdivided in pre-specified age groups of children and adolescents <18 years, patients with CHD between 18 and 65 years, and patients >65 years of age.

In-hospital information, such as treatment in an intensive care unit (ICU), invasive ventilation, resuscitation, and circulatory support, were additionally assessed. Multivariable logistic regression analysis was performed to identify the potential predictors of 30-day mortality rate. The study design is illustrated in Figure S1 of the online supplement.

### 2.2. Statistical Analysis

Categorical variables are presented as numbers and percentages, while continuous variables are presented as median and first and third quartiles (Q1 and Q3). Differences between groups were assessed using the Mann–Whitney U test or Fisher exact test depending on the data type. To investigate the association between age, gender, complexity, pre-existing heart failure, arterial hypertension, chronic lung diseases, and 30-day mortality rate, multivariable logistic regression analysis was performed, and the findings were summarized as odds ratios (OR) with respective 95% confidence intervals (CI). All analyses were explorative, and a 2-sided *p*-value of less than 0.05 was considered significant throughout the study. All analyses were performed using the R statistical software Version 4.2.0 (R Foundation for Statistical Computing, Vienna, Austria).

## 3. Results

Overall, 403 (0.5%) of the total of 83,276 patients with CHD registered in the administrative database presented in hospital with COVID-19 in 2020. Of those, 338 patients with CHD (83.9%) were diagnosed with COVID-19 infection only, while 65 (16.1%) presented with COVID-19 and associated pneumonia.

**Figure 1:**
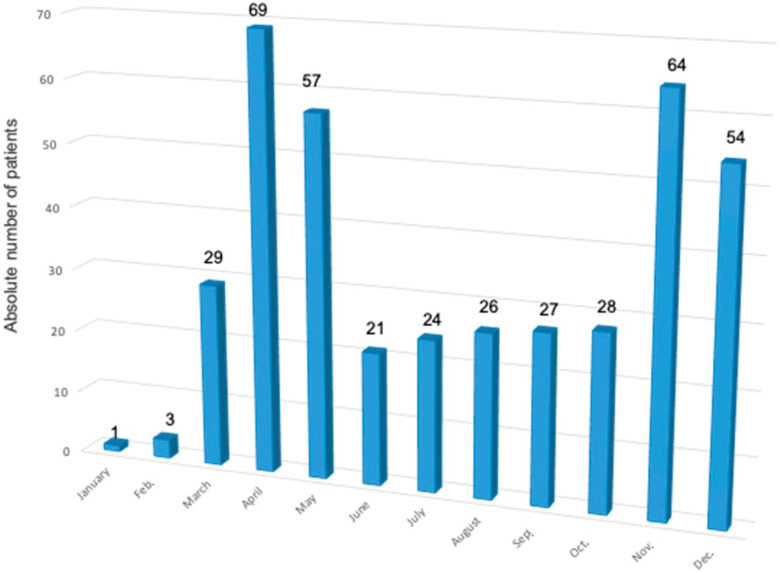
Absolute numbers of infected congenital heart disease patients throughout 2020 displayed per month. The number of patients is given above the respective block.

Figure 1 shows the absolute numbers of patients with CHD with COVID-19 throughout 2020. Two waves of infections were documented. One wave can be seen in April and May 2020, in which 31.3% (*n* = 126) of all the COVID-19 infections throughout 2020 were documented. The second wave can be observed in November and December, where 29.3% (*n* = 118) of all the infections among the patients with CHD occurred.

**Figure 2:**
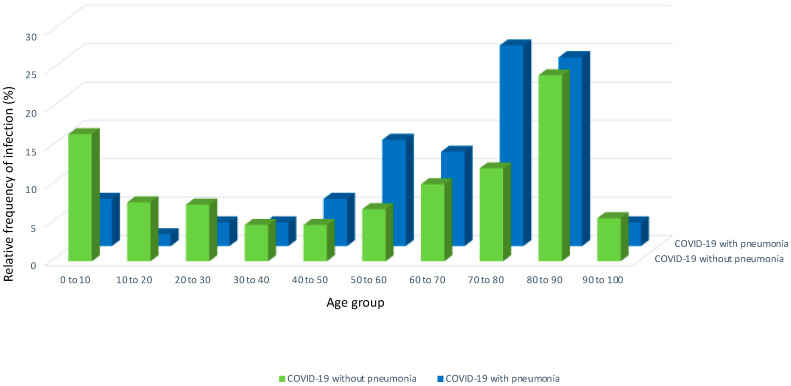
Relative frequency of congenital heart disease patients included for analysis subdivided by age in decades. The green blocks represent the relative frequency of patients with COVID-19 virus only. The blue blocks represent the relative frequency of COVID-19 patients with COVID-19 and associated pneumonia.

Figure 2 shows the relative frequency of COVID-19 virus detection only versus the patients with CHD with associated pneumonia subdivided by decades of the patients’ age. The absolute and relative numbers divided by age groups can be found in Table S3 of the online supplement.

The data show that children and adolescents were more frequently treated in a hospital for COVID-19 virus only rather than COVID-19 and associated pneumonia compared to the adults. The overall percentages were 16.6% (*n* = 56) in the children between 0 and 10 years of age and 7.7% (*n* = 26) in the patients with CHD between 10 and 20 years with COVID-19 virus only. By contrast, associated pneumonia occurred in only 6.2% (*n* = 4) of the children between 0 and 10 and 1.5% (*n* = 1) of the adolescents at 10–20 years of age. In the patients with CHD older than 40 years, the ratio reversed so that relatively more patients presented with associated pneumonia than with the virus only. For example, in the patients between 70 and 80 years, 12.1% (*n* = 41) presented with COVID-19 virus only, whilst 26.2% (*n* = 17) presented with associated pneumonia. Correspondingly, unadjusted analysis revealed a significant age difference between the patients with COVID-19 virus only (62.1 years (interquartile range (IQR) 21.7–81.3) compared to those with associated pneumonia (72.6 years (IQR 57.3–80.3) (*p* = 0.04). Additional demographic information of the study cohorts can be found in Table 1, and more detailed information on the specific CHD is provided in Table S4 of the online supplement.

In addition to the significant age difference between the two cohorts, cardiovascular comorbidities such as obesity (*p* = 0.06) were more frequently observed in the patients that presented with pneumonia compared to the patients with the diagnosis of COVID-19 only.

Table 2 gives detailed information on the in-hospital treatment of the included patients.

Unadjusted analysis showed that the median length of in-hospital stay (median 5 days versus median 8 days, *p* = 0.01) and median costs (median EUR 3524 versus median EUR 4076, *p* = 0.02) were markedly lower in the patients with COVID-19 virus only compared to those with associated pneumonia. The median costs and length of in-hospital stay further increased with the age of the affected patient. Also, the numbers of patients treated on an ICU (4.4% versus 12.3%, *p* = 0.02) and those in need of invasive ventilation (10.4% versus 21.5%, *p* = 0.02) were significantly lower among the patients with COVID-19 virus only compared to the patients with associated pneumonia. With advancing age, the number of patients in need of treatment on an ICU increased in both the cohorts. Accordingly, no patient < 18 in the cohort with COVID-19 virus only or with associated pneumonia was treated at an ICU, whilst among the patients > 65 years, eleven patients with COVID-19 infection only and six from the pneumonia cohort were treated at an ICU. In the cohort of COVID-19 infection only, the other in-hospital data did not show a clear association with advancing age. For example, invasive ventilation was required for 13 patients < 18 years, for 8 patients between 18 and 65 years, and for 14 patients > 65 years of age. In the group of patients with COVID-19-associated pneumonia, however, a tendency for the increased use of invasive ventilation with advancing age was observed. Thus, one patient < 18 required invasive ventilation compared to three patients between 18 and 65 years and ten patients > 65 years.

Table 3 includes descriptive data of all the patients who died within 30 days after the diagnosis of COVID-19 infection or associated pneumonia, respectively.

Of the patients with CHD who were diagnosed with COVID-19 infection, 5.5% (*n* = 22) died within 30 days after diagnosis. Of the 65 patients with associated pneumonia, 21.5% (*n* = 14) died. The median age of patients who died with COVID-19 virus was 82.6 years. The median age of the patients with associated pneumonia (median age 81.9 years) differed only marginally from the patients without pneumonia (*p* = 0.67). Overall, no child or adolescent died within 30 days after COVID-19 diagnosis in our dataset.

**Figure 3:**
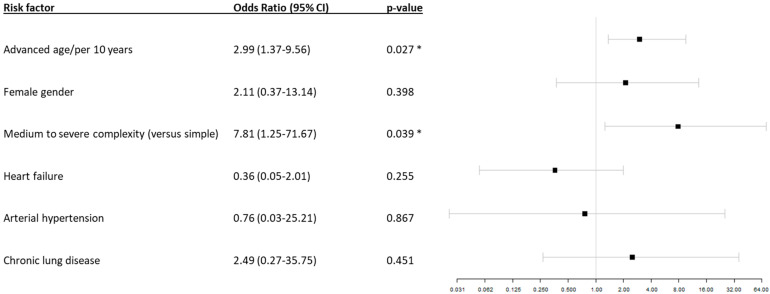
Time-dependent multivariable logistic regression model for 30-day mortality rate after diagnosis of COVID-19. Significant differences (*p* < 0.05) of the odds ratios are marked with *.

To identify the predictors of mortality in all the patients included in the analysis (COVID-19 only and associated pneumonia), time-dependent multivariable logistic regression analysis was performed, which is displayed in Figure 3.

The analysis showed that advancing age (in decades) was independently associated with an increased risk of death within 30 days in the patients with CHD (odds ratio (OR) 2.99 (95% confidence interval (CI) 1.37–9.56, *p* = 0.027). Furthermore, the patients with moderate-to-complex CHD were found to be at an increased risk (OR 7.81, 95% CI 1.25–71.67, *p* = 0.039). Other parameters, such as the female sex (OR 2.11, 95% CI 0.37–13.14, *p* = 0.398), heart failure (OR 0.36, 95% CI 0.05–2.01, *p* = 0.255), or chronic lung disease (OR 2.49, 95% CI 0.27–35.75, *p* = 0.451), were not independently associated with the 30-day mortality rate.

## 4. Discussion

Our administrative inpatient data demonstrate that COVID-19 infection with pneumonia mainly affected elderly patients with congenital heart disease rather than children and adolescents. Advanced age was, moreover, independently associated with mortality. Although the complexity of congenital heart disease emerged as a significant predictor for mortality, the overall advanced age of the deceased patients (>80 years) suggests that especially age was a critical parameter for adverse outcomes in the COVID-19 pandemic. Consequently, although patients with congenital heart disease must be perceived as a population at risk, the age of the patients with congenital heart disease should be specifically acknowledged and involved in the decision-making process regarding preventive measures and treatment.

Our data show that the number of patients with CHD affected by COVID-19 was overall relatively low: 0.5%. The unselected data of the general population collected by the German Robert Koch Institute revealed an overall number of COVID-19 infections in 2020 in Germany of 1,748,644 out of 83.2 million inhabitants, corresponding to a relative rate of 2.1% [13]. The cause of the generally lower incidence of infection in the currently presented data on patients with CHD compared to that of the general population has been observed before in an observational study based on a survey and may be explained by the advocated preventive measures for risk groups and the resulting stricter avoidance of social occupational activities [8,14,15]. Nevertheless, the social and psychological consequences of strict isolation measures must be considered when selecting risk groups in future pandemics. The patients with CHD are already disadvantaged compared to the general population in terms of career and social development, even in non-pandemic times [16,17,18]. Consequently, strict isolation measures—these may be required from an epidemiological standpoint—may further isolate this patient cohort that is relatively young compared to the general population. As discussed earlier, the negative effects of isolation measures particularly affect young patients, which further emphasizes this point [11]. Our data cannot and do not aim to answer the question regarding whether the imposed health policy measures were adequate in hindsight. By highlighting the pattern of complications observed in this early disease era affecting the previously unexposed and unvaccinated patients with CHD, we merely aim to assist informed decision making and the valuation of the approach implemented.

When focusing on the ratio of patients with CHD with COVID-19 versus those with associated pneumonia, it becomes apparent that a minority of the patients with CHD suffered from pneumonia. Although the other symptoms that were not queried in this analysis may have been present, the low incidence of pneumonia suggests that most infections were detected by chance in the patients presenting with other medical conditions through the screening programs implemented in the health care system countrywide. This is in accordance with other analyses that showed that the majority of patients with COVID-19 disease and CHD are asymptomatic [19]. However, the age structure of the analyzed cohorts is of particular importance. Whilst the relative number of patients with associated pneumonia versus the virus only was high in the older patients with CHD, the children and adolescents appeared to be more frequently infected, but less frequently suffered a severe course. Age was also identified as a strong predictor for mortality in COVID-19 independently of associated pneumonia. The perception that age plays an important role is supported further by the advanced median age of the deceased patients of 82.6 and 81.9 years and those with and without pneumonia, respectively. The association between age and morbidity or mortality in COVID-19 patients has been observed for non-congenital patients previously [20,21,22]. Other data on patients with CHD point to a similar direction, but the previous results remain, in part, conflicting [23,24]. Our data seem to support the notion that the age of the patients plays a particularly important role in unvaccinated patients with CHD.

Cardiovascular comorbidities tended to be more prevalent in the pneumonia cohort, but there was no single cardiovascular comorbidity that could be identified as a specific predictor of mortality. The overrepresentation of cardiovascular comorbidities in the pneumonia group, however, indicates that these patients maybe more prone to a symptomatic course of infection. Our results are in agreement with an analysis by Sabatino et al., showing that patients with CHD with cardiovascular comorbidities were specifically at risk of adverse outcomes [15]. Although speculative, the fact that no specific cardiovascular comorbidity could be identified as a risk factor in this analysis might point to an additive effect of comorbidities combined with the overall limited number of events in our study. The data from patients without CHD indicate, however, that the key determinants for COVID-19-associated pneumonia are age and cardiovascular comorbidities [1,4,25]. Furthermore, the prior data indicate that cardiovascular events can occur as a consequence of COVID-19 infection [4]. These were not assessed in this analysis.

Another predictor of mortality in patients with CHD infected with COVID-19 was the complexity of CHD in our analysis. The patients with CHD, and specifically, those with other than mild CHD are known to show relatively high morbidity and mortality rates in the most clinical infectious scenarios [5,26]. In this analysis, the patients with complex CHD were specifically at risk of a fatal outcome after COVID-19 infection. The OR for the 30-day mortality rate was 7.8 compared to simple CHD, albeit with a broad confidence interval. The previous analyses based on data from specialized centers, however, showed no association between severe COVID-19 infection or a fatal outcome with the anatomic complexity of CHD, but with specific lesions, such as cyanotic heart disease or pulmonary hypertension, among others [19,24,27]. Therefore, beyond the anatomic complexity of CHD, poor physiological stages (e.g., cyanosis) may lead to unfavorable outcome in the case of COVID-19 infection [28]. Overall, these analyses indicate that individual patient evaluation is essential in preventive and therapeutic decision making for patients with CHD. Potentially, specifically in the younger patients with CHD that have shown to be the least affected clinically, but most severely impaired by preventive measures, the complexity of disease and physiological state may aid in deciding in these cases.

### Limitations

This analysis is based on administrative data. As these data were primarily gathered for reimbursement purposes, the data are complete for all individuals and reflect a nationwide unselected cohort. However, there are general constraints in using administrative data for analyses, which have been described before [29]. Coding errors may be present. These data give an overview over all the inpatient cases of patients with CHD with COVID-19, but the cause of in-hospital admission is not known. Specifically, high-risk patients may have been missed in this analysis, as they may have avoided hospital medical contact out of fear of further infection. Furthermore, it is not possible assess the laboratory values and clinical parameters. While the death data are included in the underlying database, the cause of death was not documented. We cannot completely exclude the possibility that COVID-19-related mortality cases in children were missed, and the mortality rate in this population might have been underestimated. We contend that it is highly unlikely that young patients with CHD died at home of COVID-19-related complications without being first admitted to hospital. However, it appears possible that for the selected patients with CHD in a terminal or palliative setting, the treating physicians may have refrained from hospital admission. All the analyses presented represent associations, and we cannot comment on causation. In particular, the need for invasive ventilation does not necessarily indicate a severe course of COVID-19 infection, as patients with CHD may also be permanently dependent on invasive ventilation. This may explain why more patients were in need of invasive ventilation than those treated on an ICU. The data presented apply specifically to the year 2020 in a high-resource health care system and cover only approximately 1/9 of the German population. This time window was chosen as no COVID-19 vaccination was available before 2021 in Germany. Therefore, all the results should be applied to patients with CHD who were unexposed to COVID-19 and were unvaccinated. The current cohorts of patients are mostly vaccinated, and even those who refused appropriate vaccines will have most likely been in contact with the virus. Therefore, specific care should be taken when applying the results of our analysis to current and future CHD cohorts. The current data cannot be extrapolated to community-based ambulatory patients.

## 5. Conclusions

COVID-19 infection was associated with pneumonia, specifically in elderly patients with congenital heart disease. Advanced age was, moreover, independently associated with mortality. More-than-mildly complex congenital heart disease emerged as an additional predictor of adverse outcomes. Consequently, age and the complexity of congenital heart disease should be regarded as the critical parameters for adverse outcomes in unvaccinated patients with congenital heart disease in the early era of the COVID-19 pandemic.

## Figures and Tables

**Table 1:** Patient demographics of congenital heart disease patients (CHD) stratified into those with diagnosis of COVID-19 only and additional pneumonia. Additional subdivision of patients into patients < 18 years and 18–65 years and >65 years of age. The given *p*-value compares patients with COVID-19 infection to patients with associated pneumonia.

	Patients with COVID-19 without Pneumonia				Patients with COVID-19 and Pneumonia				*p*-Value
Median age (Q1, Q3)	62.1 (21.7, 81.3)				72.6 (57.3, 80.3)				0.04
Age group	All	<18	18–65	>65	All	<18	18–65	>65	
Number of patients	338	75	103	160	65	4	23	38	
Female sex	184 (54.4)	32	56	96	29 (44.6)	2	10	17	0.18
Congenital heart disease (CHD)									
Simple CHD, *n* (%)	243 (71.9)	49	70	124	48 (73.8)	3	17	28	0.88
Moderate CHD, *n* (%)	52 (15.4)	11	20	21	12 (18.5)	1	5	6	0.58
Severe CHD, *n* (%)	43 (12.7)	5	13	15	5 (7.7)	0	1	4	0.05
Other patient characteristics									
Coronary artery disease, *n* (%)	65 (19.2)	0	9	56	19 (29.2)	0	5	14	0.09
Congestive heart failure > NYHA II, *n* (%)	89 (26.3)	6	18	65	14 (21.5)	0	4	10	0.54
Diabetes mellitus, *n* (%)	107 (31.7)	1	22	84	28 (43.1)	0	9	19	0.09
Chronic kidney disease, *n* (%)	123 (36.4)	1	27	95	28 (43.1)	0	7	21	0.33
Obesity, *n* (%)	109 (32.3)	4	32	73	29 (44.6)	0	13	16	0.06
Nicotine abuse, *n* (%)	59 (17.5)	0	27	32	15 (23.1)	0	5	10	0.30
History of stroke, *n* (%)	79 (23.4)	1	18	60	27 (41.5)	0	6	21	0.003
Cancer, *n* (%)	143 (42.3)	2	30	111	34 (52.3)	0	8	26	0.17
Chromosomal anomaly, *n* (%)	20 (5.9)	8	11	1	6 (9.2)	1	5	0	0.40
Drug therapy of selective cardiac and non-cardiac drugs									
Oral anticoagulants, *n* (%)	118 (34.9)	3	28	87	29 (44.6)	0	6	23	0.16
Platelet activation inhibitor, *n* (%)	117 (34.6)	7	22	88	28 (43.1)	0	7	21	0.21
Statins, *n* (%)	133 (39.3)	0	24	109	37 (56.9)	0	7	30	0.009
Beta blocker, *n* (%)	185 (54.7)	1	47	137	44 (67.7)	1	14	29	0.06
ACE- inhibitors/Angiotensin II receptor blockers, *n* (%)	194 (57.4)	4	47	143	46 (70.8)	0	12	34	0.05
Diuretics, *n* (%)	180 (53.3)	10	38	132	42 (64.6)	0	12	30	0.10
Non-steroidal antirheumatic agents, *n* (%)	282 (83.4)	50	89	143	55 (84.6)	0	20	35	1

**Table 2:** Details on in-hospital treatment of congenital heart disease patients comparing patients with infection only compared to patients with associated pneumonia. Patients are additionally subdivided by age. The given *p*-value compares patients with COVID-19 infection to patients with associated pneumonia.

	Patients with COVID-19				Patients with COVID-19 and Pneumonia				*p*-Value
Age	All	<18	18–65	>65	All	<18	18–65	>65	
Median length of in-hospital stay, days (Q1, Q3)	5 (3, 12)	3 (2, 8.5)	4 (2.5, 8.5)	8 (4, 16)	8 (5, 14)	3.5 (3, 8)	7 (5, 11.5)	10 (4, 15)	0.01
Median costs, EUR (Q1, Q3)	3524 (2434, 6843)	2726 (2078, 6922)	3253 (2442, 5474)	4094 (2800, 7266)	4076 (3151, 7078)	3041 (2886, 4335)	3799 (3180, 6734)	4349 (3102, 9104)	0.02
Treatment at intensive care unit, *n* (%)	15 (4.4)	0	4	11	8 (12.3)	0	2	6	0.02
Invasive ventilation, *n* (%)	35 (10.4)	13	8	14	14 (21.5)	1	3	10	0.02
Circulatory support, *n* (%)	8 (2.4)	6	2	0	1 (1.5)	0	1	0	1
Resuscitation, *n* (%)	12 (3.6)	1	6	5	3 (4.6)	0	1	2	0.72

**Table 3:** Patient characteristics including all patients that died within 30 days after COVID-19 detection. Patients are additionally subdivided by age. The given *p*-value compares patients with COVID-19 infection to patients with associated pneumonia. No patient < 18 years of age died.

	Patients with COVID-19 Only (*n* = 22)			Patients with COVID-19 and Pneumonia (*n* = 14)			*p*-Value
Median age–years (Q1, Q3)	82.6 (77.5, 88.7)			81.9 (77.0, 83.4)			0.67
Age group	All	18–65	>65	All	18–65	>65	
Female sex, *n* (%)	11 (50.0)	1	10	7 (50.0)	0	7	1
Congenital heart disease							
Simple, *n* (%)	17 (77.3)	1	16	7 (50.0)	0	7	0.15
Moderate, *n* (%)	2 (9.1)	1	1	3 (21.4)	0	3	0.36
Severe, *n* (%)	3 (13.6)	0	3	4 (28.6)	1	3	0.39
Details on in-hospital treatment							
Median length of in hospital stay, days (Q1, Q3)	9 (6, 10.75)	9.5 (9.25, 9.75)	9 (5.3, 11.0)	4.5 (3.3, 10.8)	5 (5,5)	4 (3.0, 11.0)	0.43
Median costs, EUR (Q1, Q3)	3946 (3193, 6331)	4006 (3710, 4301)	3946 (3097, 6611)	3896 (3026, 12,753)	18,144 (18,144, 18,144)	3631 (2959, 11,466)	0.71
Treatment at intensive care unit, *n* (%)	1 (4.6)	0	1	2 (14.3)	0	2	0.55
Invasive ventilation, *n* (%)	2 (9.1)	0	2	5 (35.7)	1	4	0.08
Circulatory support, *n* (%)	0 (0)	0	0	1 (7.1)	1	0	0.39
Resuscitation, *n* (%)	2 (9.1)	1	1	3 (21.4)	1	2	0.36

## Data Availability

The datasets presented in this article are not readily available because insurance data are protected by the German data protection laws (‘Bundesdatenschutzgesetz’, BDSG). As for data protection laws, the uncensored data cannot be made available in the manuscript, the supplemental files, or in a public repository. They are stored on a secure drive in the Barmer health insurance research institute to facilitate replication of the results. Generally, access to data of statutory health insurance companies for research purposes is possible only under the conditions defined in German Social Law (SGB V § 287). Requests for data access can be sent as a formal proposal specifying the recipient and purpose of data transfer to the appropriate data protection agency. Access to the data used in this study can only be provided to external parties under the conditions of the cooperation contract of this research project and after written approval by the health insurance fund.

## Supplementary Materials

The following supporting information can be downloaded at: https://www.mdpi.com/article/10.3390/jcm13051282/s1, Figure S1: Study design; Table S1: ICD-10-GM Codes/OPS diagnosis and procedure codes and ATC codes relevant for analyses; Table S2: ICD-10 GM-Codes used for identification and grouping of patients with congenital heart disease (CHD); Table S3: Absolute and relative numbers of patients with congenital heart disease and COVID-19 subdivided in infection only and associated pneumonia; Table S4: Detailed information on the underlying congenital heart disease of included patients stratified in those with diagnosis von COVID-19 only and additional pneumonia. Additional stratification of patients into patients younger than 18 years and 18–65 years of age. The given p-value compares patients with COVID-19 infection to patients with associated pneumonia.
