# Supplementary material for: Impact of COVID-19 Infections among Unvaccinated Patients with Congenital Heart Disease: Results of a Nationwide Analysis in the First Phase of the Pandemic
Source: J Clin Med. 2024 Feb 24;13(5):1282. doi: 10.3390/jcm13051282 (PMC10931600; doi:10.3390/jcm13051282)
Supplement: Supplementary file 1 [file jcm-13-01282-s001.zip › jcm-2823407-supplementary.pdf]

Supplementary online only material

Table S1: ICD-10-GM Codes/ OPS diagnosis and procedure codes and ATC codes relevant for analyses.

Parameter	Codes
<u>Baseline parameters</u>	
**Arterial hypertension**	ICD: I10-I15
**Cancer**	ICD: C
**Chromosomal anomalies**	ICD: Q90, Q91-Q94, Q95-Q99
**Chronic kidney disease**	ICD: N18, N19
**Chronic lung disease**	ICD: J40-J47
**Congestive heart failure (CHF) symptoms**	ICD:
**NYHA III**	I50.13
**NYHA IV**	I50.14
**Coronary artery disease**	ICD: I25.11, I25.12, I25.13
**Diabetes mellitus non-specified**	ICD: E10-E14
**Dyslipidemia**	ICD: E78
**Nicotine abuse**	ICD: F17
**Obesity**	ICD: E66
<u>Drugs</u>	
**ACE- inhibitors/ Angiotensin II receptor blockers (ARB)**	ATC: C09A-D
**Betablockers**	ATC: C07
**Diuretics**	ATC: C03
**Immunosuppressants**	ATC: L04A, H02AB, H02B
**Non-steroidal anti-inflammatory drugs (NSAID)**	ATC: C01EB16, M01AE01, C01EB16, M01A
**Oral anticoagulants**	ATC: B01AA, B01AE07, B01AF (from 2013), B01AX06 (only 2012), B01AX08 (only 2012)
**Platelet activation inhibitor (PAI)**	ATC: B01AC
**Statin**	ATC: C10AA, C10BA, C10BX
<u>In-hospital parameters</u>	
**Circulatory support**	OPS: 5-375, 5-376, 5-37b, 8-851, 8-852, 8-83a, 8-839.4, 8.839.a, 8.839.b
**Death**	ICD: A40.x, A41.x
**Invasive ventilation**	OPS: 8-70, 8-71, 5-311
**Resuscitation**	OPS: 8-771
**Treatment on the intensive care unit**	OPS: 8-980

Table S2: ICD-10 GM-Codes used for identification and grouping of patients with congenital heart disease (CHD).

Parameter	Codes
**<u>Simple CHD</u>**	
**Isolated congenital valve disease**	ICD: Q23.0, Q23.1, Q22.4, Q22.8, Q22.9, Q23.2, Q23.3, Q22.1, Q22.2, Q22.3
**Isolated ventricular septal defect**	ICD: Q21.0
**Other congenital malformation of the great arteries**	ICD: Q25.8, Q25.9
**Persistent arterial duct**	ICD: Q25.0
**<u>Moderate complexity CHD</u>**	
**Aortic isthmus stenosis, interrupted aortic arch**	ICD: Q25.1, Q25.2
**Atrioventricular septal defect**	ICD: Q21.2
**Ebstein's anomaly**	ICD: Q22.5
**Partial anomalous pulmonary venous connection**	ICD: Q26.3, Q26.4
**Tetralogy of Fallot**	ICD: Q21.3, Q21.80, (Q22.0 and Q21.0)
**<u>Severe CHD</u>**	
**Eisenmenger's syndrome**	ICD: I27.8 and at least one further Q-Code except for Q21.1<br>Or Q21.88 and at least one futher Q-Code except for Q21.1
**Univentricular heart**	ICD: Q20.1, Q20.2, Q20.4, Q22.6, Q23.4, (Q22.0 without Q21.0)
**Transposition of the great arteries (TGA)**	ICD: Q20.3, Q20.5
**Other complex heart malformation, e.g. total anomalous pulmonary venous connection, common arterial trunk**	ICD: Q20.0, Q26.2

Figure S1 Study design.

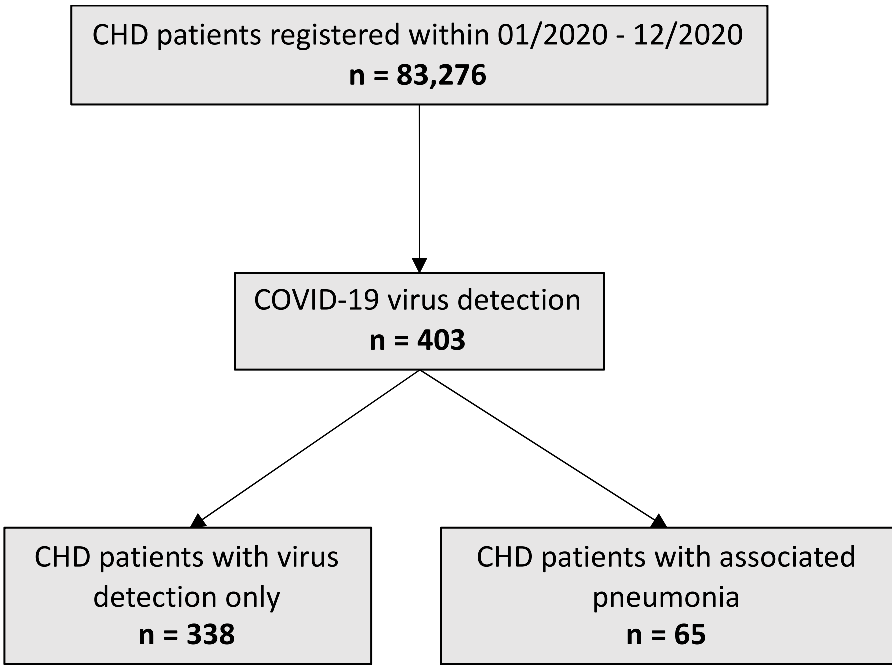

Table S3 Absolute and relative numbers of patients with congenital heart disease and COVID-19 subdivided in infection only and associated pneumonia.

Age group (in decades)	Patients with COVID-19 (n=338)	Patients with COVID-19 and pneumonia (n=65)
**0 to 10, n (%)**	56 (16.6)	4 (6.2)
**10 to 20, n (%)**	26 (7.7)	1 (1.5)
**20 to 30, n (%)**	25 (7.4)	2 (3.1)
**30 to 40, n (%)**	16 (4.7)	2 (3.1)
**40 to 50, n (%)**	16 (4.7)	4 (6.2)
**50 to 60, n (%)**	23 (6.8)	9 (13.8)
**60 to 70, n (%)**	34 (10.1)	8 (12.3)
**70 to 80, n (%)**	41 (12.1)	17 (26.2)
**80 to 90, n (%)**	82 (24.3)	16 (24.6)
**90 to 100, n (%)**	19 (5.6)	2 (3.1)

Table S4 Detailed information on the underlying congenital heart disease of included patients stratified in those with diagnosis von COVID-19 only and additional pneumonia. Additional stratification of patients into patients younger than 18 years and 18 – 65 years of age. The given p-value compares patients with COVID-19 infection to patients with associated pneumonia.

	**Patients with COVID-19 (n=338)**				**Patients with COVID-19 and pneumonia (n=65)**				**p-value**
**Age group**	**all**	**<18**	**18-65**	**>65**	**all**	**<18**	**18-65**	**>65**	
- Isolated ventricular septal defect	72 (21.3)	21	19	32	13 (20.0)	2	7	4	0.87
- Persistent arterial duct	27 (8.0)	22	1	4	4 (6.2)	1	0	3	0.80
- Isolated congenital valve disease	141 (41.7)	6	49	86	28 (43.1)	0	9	19	0.89
- Other congenital malformation of the great arteries	3 (0.9)	0	1	2	3 (4.6)	0	1	2	0.06
Moderate CHD – n (%)									
- Tetralogy of Fallot	6 (1.8)	2	4	0	1 (1.5)	0	0	1	1
- Ebstein's anomaly	2 (0.6)	0	1	1	0 (0)	0	0	0	1
- Aortic isthmus stenosis, interrupted aortic arch	18 (5.3)	4	6	8	6 (9.2)	0	3	3	0.25
- Atrioventricular septal defect	23 (6.8)	4	9	10	5 (7.7)	1	2	2	0.8
- Partial anomalous pulmonary venous connection	3 (0.9)	1	0	2	0 (0)	0	0	0	1
Severe CHD – n (%)									
- Univentricular heart	14 (4.1)	9	2	3	2 (3.1)	0	1	1	1
- Eisenmenger's syndrome	17 (5.0)	1	5	11	2 (3.1)	0	0	2	0.75
- Transposition of the great arteries (TGA)	8 (2.4)	3	4	1	0 (0)	0	0	0	0.36
- Other complex heart malformation	4 (1.2)	2	2	0	1(1.5)	0	0	1	0.59
